# Optimizing unsupervised clustering of electrochemical impedance spectra via normalization and dimensionality reduction

**DOI:** 10.1038/s41598-026-35621-3

**Published:** 2026-01-20

**Authors:** Sanja Martinez, Ines Bera, Izabela Martinez, Ivana Šoić

**Affiliations:** 1 Faculty of Chemical Engineering and Technology, University of Zagreb, Marulićev trg 19, Zagreb, 10000 Croatia; 2 Faculty of Mechanical Engineering and Naval Architecture, University of Zagreb, Ivana Lučića 5, Zagreb, 10002 Croatia

**Keywords:** Chemistry, Engineering, Materials science, Mathematics and computing

## Abstract

Electrochemical impedance spectroscopy (EIS) is entering an exciting stage of development as machine learning–driven (ML) spectral analysis begins to complement traditional equivalent-circuit fitting and address some of its practical limitations. The appeal of a simple, fully data-driven, unsupervised workflow is clear: by operating directly on EIS spectra, it bypasses the additional modelling layers required for equivalent-circuit fitting, handcrafted feature extraction, or supervised training. Here we demonstrate that normalization and dimensionality reduction play a critical, yet previously overlooked, role in shaping the outcomes of unsupervised workflows. Using welded stainless steel as a demonstrator, we systematically evaluate combinations of normalization strategies and dimensionality-reduction pipelines. By applying internal clustering metrics and a Borda ranking, we identify an effective workflow configuration, an appropriate cluster number, and a cluster structure consistent with mechanistic expectations for the studied dataset. Mechanistically anchored linear projections further rank relative passivity across the stainless-steel passivity range via k-level clustering, while bootstrap resampling confirms high cluster stability despite the modest sample size.

## Introduction

Dimensionality reduction is emerging as a promising approach for classifying high-dimensional electrochemical impedance spectra (EIS) without the need for prior labelling. The rationale is the same as for more complex machine-learning workflows: traditional analysis by equivalent-circuit fitting or handcrafted feature extraction is subject to human bias and becomes increasingly impractical for automated applications. The appeal of the simpler approach is that it avoids additional modelling layers, making the workflow transparent, computationally efficient, and easier to adopt in practice. Recent work by Makogon and co-workers^1^ addressed this approach directly by asking whether dimensionality reduction can separate spectra of different mechanistic origin without supervision. Using a synthetic database of more than 9,000 spectra from 18 representative equivalent circuits, they found that non-linear methods such as t-distributed stochastic neighbour embedding (t-SNE) and uniform manifold approximation and projection (UMAP) revealed distinct groupings, whereas principal component analysis (PCA) gave overlapping projections. However, they also noted difficulties in separating analogous cases such as capacitors and constant phase elements.

Except for this proof-of-concept, PCA has been applied to experimental EIS only sparingly: in food science^2–7^, biomedical sensing^8–10^, and coatings/materials^11–14^. In battery studies, the most advanced ML applications to EIS to date, PCA is typically limited to preprocessing for supervised modelling^15–17^, indicating that its unsupervised potential remains largely unexplored. Beyond PCA, other dimensionality reduction methods are beginning to appear. Chen et al.^18^ applied t-SNE to time-lapse EIS during bacterial growth, while autoencoder/variational autoencoder embeddings^19–24^ have been used in batteries, and classical multidimensional scaling^25^ has been applied to beverage EIS clusters. UMAP, by contrast, has so far only been tested on synthetic data^1^, with no experimental reports.

In the present study, experimental EIS spectra of welded stainless steel are used as a demonstrator. Applications of unsupervised analysis to electrochemical data of passive alloys have only very recently appeared. Coelho et al.^27^ applied PCA and unsupervised clustering to polarization curves of 316 L stainless steel, identifying distinct pitting pathways but without using EIS. Kurtz and co-workers^28^ clustered impedance-derived parameters obtained from equivalent-circuit fitting, providing the only example of unsupervised clustering applied to EIS data of passive metals. In parallel, the same group trained deep neural networks in a supervised fashion on near-field spectra^29^ to classify dissolution states in Ti-6Al-4 V bioimplants. A recent review^30^ further emphasized the promise of AI for predicting corrosion in orthopaedic biomaterials, while highlighting the scarcity of basic science studies applying machine learning to electrochemical datasets.

In summary, only a small number of truly data-driven, unsupervised studies of EIS exist and the impact of normalization and dimensionality reduction on those workflows has not been systematically investigated. Across the literature, raw EIS data normalization prior to dimensionality reduction is handled in a variety of ways, including per-sample normalization^1,13,14^, autoscaling^2,5,11^, or reliance on software defaults^7^. The recent study by Sun et al.^26^ underscored the importance of normalization in the context of neural networks, systematically comparing different data representations and normalization methods. Here we address this gap for fully unsupervised workflows, applying normalization and dimensionality reduction directly to raw experimental EIS spectra. We show that the choice of normalization and dimensionality-reduction method critically shapes separability in reduced-dimensional space. Using mechanistically anchored linear projections with k-level clustering on well separated data, we positioned material states relative to reference subgroups, enabling stable unsupervised EIS interpretation.

In this work, we use the term “analysis” strictly in the context of unsupervised structure discovery, not mechanistic electrochemical interpretation. The workflow identifies groups of spectra with similar geometric features in reduced-dimensional space, but it does not substitute for modelling approaches such as equivalent-circuit fitting, which extract physical parameters and explain why impedance responses change. We therefore present our approach as a complementary classification and visualization tool that supports, rather than replaces, traditional electrochemical analysis. Physics-informed interpretation of the discovered structures is outside the scope of the present work, but represents a natural direction for extending the workflow in future studies.

## Dimensionality reduction workflow design

**Fig. 1:**
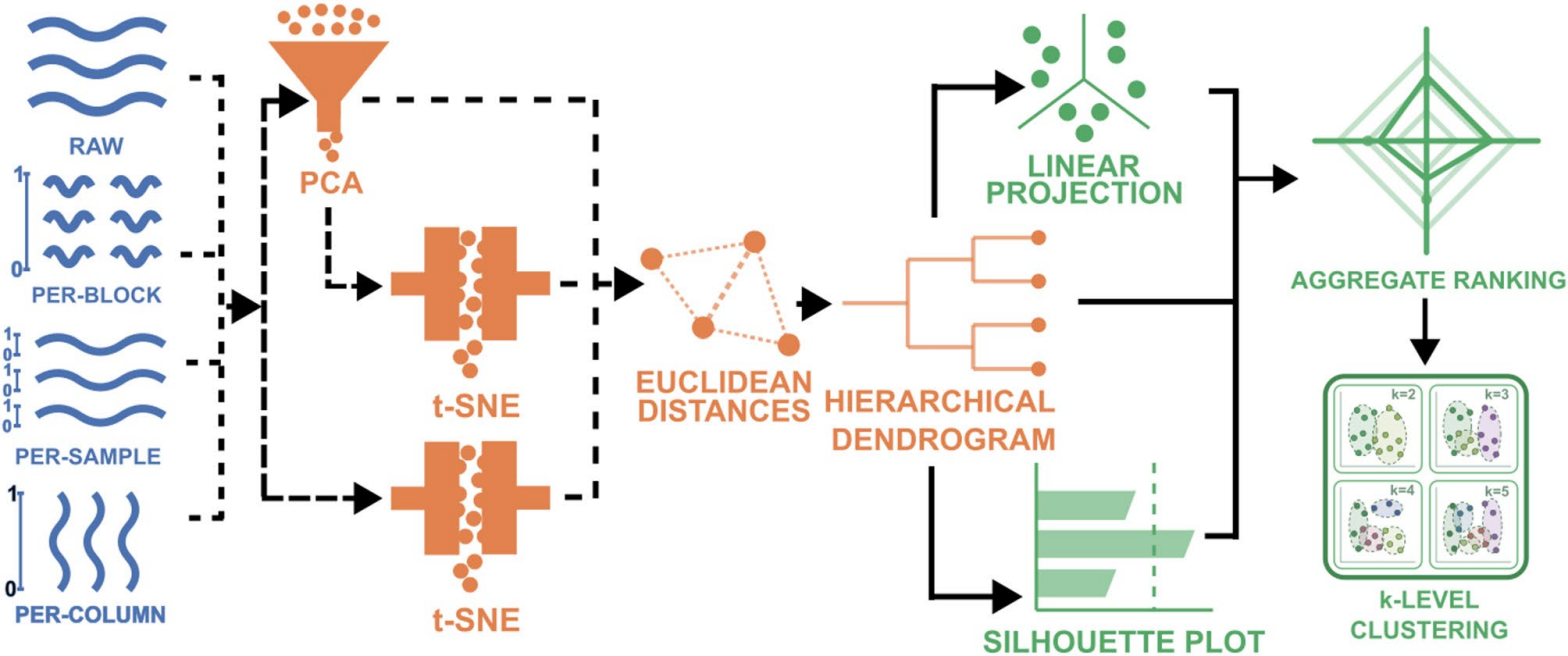
Workflow for preprocessing, dimensionality reduction, and clustering of EIS spectra. Impedance spectra are preprocessed using one of four schemes (raw, per-block min–max or per-sample min–max normalization or per-column autoscaling), then embedded via PCA, t-SNE, or PCA + t-SNE. Euclidean distances in the reduced space feed hierarchical clustering (dendrogram). Cluster structure is visualized with a reference-anchored linear projection. Clustering results are ranked by Borda count using clustering validity indices (silhouette score, Davies–Bouldin, Calinski–Harabasz), with silhouette plots used for visual inspection. The linear projection positions spectra along a continuum between chosen mechanistic references, enabling structure discovery by comparing clusters to mechanistic subgroups. Dashed arrows indicate alternative routes.

The dimensionality reduction workflow (Fig. 1) begins with normalization of the impedance spectra.

Furthermore, we consider two variants: (i) per-block normalization, where all log|Z| values are divided by the dataset-wide maximum log|Z| and all phase values by the dataset-wide maximum of phase, using robust scaling bounds rather than strict extrema, and (ii) per-sample normalization applied separately to log|Z| and to phase, whereby within each spectrum the log|Z| is rescaled to [0,1] from its own minimum and maximum and the phase is independently rescaled to [0,1].

For per-block normalization, log|Z| and phase were treated as separate data blocks, *M* and *P*.

For a given block $X \in \left\{ {M,P} \right\}$, percentile bounds $\left( {\alpha =1,~\beta =99} \right)$ were computed over all samples and frequencies:

$${\ell _X}={\mathrm{per}}{{\mathrm{c}}_\alpha }\left( X \right),{u_X}={\mathrm{per}}{{\mathrm{c}}_\beta }\left( X \right)$$

where ${\mathrm{per}}{{\mathrm{c}}_p}\left( X \right)$is the *p*-th percentile computed over all finite entries of block *X*. Percentile bounds (α = 1, β = 99) ensure that the central 98% of values define the normalization range, while extreme outliers do not influence the scaling bounds.

Scaling bounds were then obtained from a clipped auxiliary array:

$${X^{{\mathrm{clip}}}}={\mathrm{min}}\left( {{\mathrm{max}}\left( {X,{\ell _X}} \right),{u_X}} \right),$$

such that

$${X_{{\mathrm{min}}}}={\mathrm{min}}\left( {{X^{{\mathrm{clip}}}}} \right),{X_{{\mathrm{max}}}}={\mathrm{max}}\left( {{X^{{\mathrm{clip}}}}} \right).$$

The normalized values were computed from the original (unclipped) data as

$${X^{{\mathrm{norm}}}}=\frac{{X - {X_{{\mathrm{min}}}}}}{{{X_{{\mathrm{max}}}} - {X_{{\mathrm{min}}}}}}.$$

The clipped array was used only to define normalization bounds, while the original data were normalized without clipping; therefore, a small fraction of values may lie outside the nominal [0,1] range.

For per-block normalization, let *i* index individual spectra and *j* index frequency points. For a given spectrum *i*, we denote by ${M_{i,j}}$and ${P_{i,j}}$the log|Z| and phase values, respectively. For a given spectrum *i*and block $X \in \left\{ {M,P} \right\}$, define the spectrum-specific extrema:

$$X_{{{\mathrm{min}}}}^{{}}=\mathop {{\mathrm{min}}}\limits_{j} {X_{i,j}},X_{{{\mathrm{max}}}}^{{}}=\mathop {{\mathrm{max}}}\limits_{j} {X_{i,j}}$$

where the minimum and maximum are computed over finite values only.

If $X_{{{\mathrm{max}}}}^{{}}>X_{{{\mathrm{min}}}}^{{}}$, the normalized values are:

$$X_{{i,j}}^{{{\mathrm{norm}}}}=\frac{{{X_{i,j}} - X_{{{\mathrm{min}}}}^{{}}}}{{X_{{{\mathrm{max}}}}^{{}} - X_{{{\mathrm{min}}}}^{{}}}}$$

If fewer than two finite values are present or $X_{{{\mathrm{max}}}}^{{}} \leqslant X_{{{\mathrm{min}}}}^{{}}$, the spectrum is left unchanged. This normalization maps all valid values strictly to the interval [0,1].

Both approaches preserve overall spectral shape: per-sample normalization emphasizes relative features within a spectrum, while per-block normalization preserves comparability of magnitudes across spectra.

For comparability with some works in the literature^2,5^, we replicated SOLO©’s (Eigenvector Research, Inc.) ‘Autoscale’ by mean-centring each frequency column across samples and dividing by that column’s standard deviation (column-wise mean-centring with unit-variance scaling). After autoscaling, data at every frequency, both log|Z| and phase have a zero mean and unit variance.

For each block $X \in \left\{ {M,P} \right\}$and each column *j*:

$${\mu _{X,j}}=\frac{1}{{{n_j}}}\mathop \sum \limits_{{i \in {\mathcal{I}_j}}} {X_{i,j}},{\sigma _{X,j}}=\sqrt {\frac{1}{{{n_j}}}\mathop \sum \limits_{{i \in {\mathcal{I}_j}}} {{\left( {{X_{i,j}}{\mu _{X,j}}} \right)}^2}}$$

where ${\mathcal{I}_j}$is the set of spectra with finite ${X_{i,j}}$, and ${n_j}=\mid {\mathcal{I}_j}\mid$.

Autoscaled values are then:

$$X_{{i,j}}^{{{\mathrm{auto}}}}=\frac{{{X_{i,j}} - {\mu _{X,j}}}}{{{\sigma _{X,j}}}}$$

If fewer than two finite values are present in a column or ${\sigma _{X,j}} \leqslant 0$, that column is left unchanged. Being common in chemometrics, this approach gives equal weight to all frequencies, which can overweight noisy or weakly informative regions.

To summarize, in the present study dimensionality reduction pipelines are tested on four input variants: raw spectra, per-block min–max normalized, per-sample min–max normalized, and per-column autoscaled spectra.

After normalization, dimensionality reduction is applied using PCA^31^, t-SNE^32^, or a sequential PCA + t-SNE combination. These approaches are chosen to compare linear embeddings (PCA) with nonlinear manifold learning (t-SNE), as well as their combined use. For PCA, the number of principal components is chosen to retain a sufficiently high proportion of variance, whereas for t-SNE the feature space is inherently two-dimensional.

To provide an initial, intuitive view of data structure, linear projections are visualized in two dimensions, either by circular projection of PCA components or by t-SNE projection. These visualizations allow a first assessment of clustering tendencies and separability. This step confirms whether separability corresponds to mechanistically meaningful groups, since clustering can otherwise appear numerically strong while grouping the wrong states.

Distances between spectra are then calculated in the reduced feature space. Hierarchical clustering based on Euclidean distances is performed, and cluster quality is assessed in an unbiased fashion using aggregation-ranking by three metrics: average silhouette score^33^, Davies–Bouldin index^34^, and Calinski–Harabasz index^35^.

Finally, for the highest-quality clustering, we compute a reference-anchored linear projection that positions each spectrum on a continuum between two mechanistic reference subgroups in the reduced feature space. This anchor-based positioning enables k-level clustering and determination of relative passivity among clusters.

Robustness was assessed by stratified and unstratified bootstrapping of the dataset, enabling internal and external validation through the distributions of Adjusted Rand Index (ARI), Normalized Mutual Information (NMI), and purity^36^ across resampled subsets and mechanistic subgroups. Internal validation was done by comparing bootstrapped clustering with that of the original dataset, while in external validation agreement was investigated between clustering results and mechanistic labels.

For implementation, we used Orange^37^, an open-source data mining platform that provides widget-level code openly on GitHub. The choice of Orange was motivated by its accessibility for non-specialists and its transparency for machine learning experts. In this study, built-in widgets were combined with custom Python routines, integrated through Python Script widgets, to extend functionality.

For every normalization + dimensionality-reduction combination, the input spectra are available in the Input Data folder, the code is available in the Code folder, the resulting plots are available in the Graphs folder, and validation indices, bootstrap results and clustering scores are available in the Results folder of our OSF repository (see Data Availability). In the main text, we show only a representative subset. This balances transparency (full set online) with readability (key cases in the manuscript).

### Welded stainless steel spectra

The impedance spectra analysed in this study were obtained from our previously published dataset^38^, which is freely available under a CC-BY license. The experimental procedures for sample preparation, welding, and impedance measurements are fully described in that reference and are not repeated here, as the present work focuses on unsupervised spectral analysis. Spectra were acquired on 316L stainless steel, in the paste electrolyte cell at open-circuit potential using a three-electrode configuration and span eight mechanistic subgroups defined by surface region (base vs. heat-affected zone), and surface treatment, namely as-welded or mechanically cleaned states, each either unpassivated or citric-acid passivated. As surfaces with different preparation histories are expected to exhibit correspondingly different impedance responses, such variations appear only weakly in the present dataset and therefore do not yield robustly separable subgroups.

**Fig. 2:**
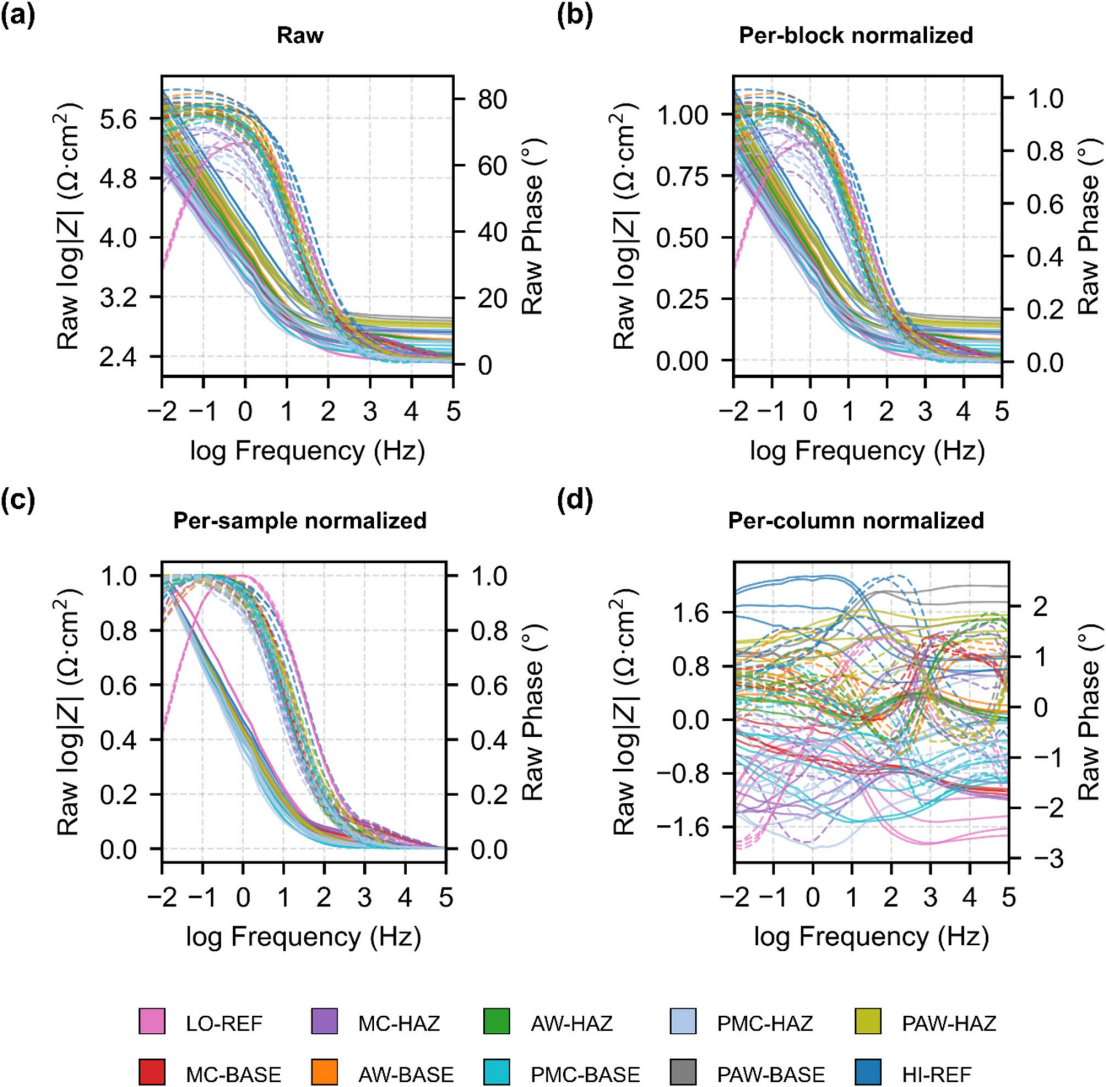
Impedance spectra for machine-learning input. Bode spectra (log|Z| and phase) measured on stainless steel 316 L samples, including base metal (BASE) and heat-affected zone (HAZ) regions in five surface states: mechanically-cleaned (MC), as-welded (AW), mechanically-cleaned and citric-acid-passivated (PMC), and as-welded and citric-acid-passivated (PAW). Spectra of abraded (ABR) and nitric-acid-passivated (NPAS) non-welded reference samples are also shown. a, raw spectra. b, per-block min–max normalized spectra, with log|Z| and phase scaled separately by the dataset-wide maximum of each block. c, per-sample min–max normalized spectra, with log|Z| and phase scaled separately by the maximum of each individual spectrum. d, per-column autoscaled spectra, with each frequency column transformed to zero mean and unit variance across the dataset. For per-block normalized spectra, scaling is anchored to robust percentiles rather than absolute extrema; as a result, a small fraction of points can exceed 1 (or fall below 0). We retain these values to avoid distorting separability.

Figure 2a shows the raw impedance spectra that serve as input to the machine-learning workflow. The raw spectra dataset is provided in the file *input_data.csv* (Input Data folder in the repository; see Data Availability). Bode plots of log|Z| and phase were collected from welded 316 L stainless steel, comparing base metal (BASE) and heat-affected zone (HAZ) regions. Samples were examined in several surface conditions: mechanically cleaned (MC), as-welded (AW), mechanically cleaned and citric-acid-passivated (PMC), and as-welded and citric-acid-passivated (PAW). Spectra of abraded (ABR) and nitric-acid-passivated (NPAS) non-welded reference samples are also shown, representing the “oxide-free” (LO-REF) and “best-passivated” (HI-REF) states used for *k*-level clustering and mechanistic analysis. The panels illustrate spectra in raw form (a) and after normalization within the workflow: (b) per-block min–max normalization, (c) per-sample min–max normalization, and (d) per-column autoscaling. The strong overlap of spectra highlights the need for dimensionality reduction to achieve separability.

### Workflow optimization

Three pipelines, PCA, t-SNE, and their sequential combination (PCA + t-SNE), were used for dimensionality reduction. The codes are provided separately in the files *PCA_workflow.ows*, *PCA_t-SNE_workflow.ows*, and *t-SNE_workflow.ows* (Code folder in the repository; see Data Availability).

PCA was applied directly to the raw or normalized spectra, with the number of retained components set to four, corresponding to a cumulative explained variance of 98%. The individual contributions depend on the pipeline used. As an example, for the later determined best-ranked pipeline, they are 59.42% (PC1), 24.12% (PC2), 12.49% (PC3), and 2.11% (PC4).

t-SNE^32^ was run via Orange’s t-SNE widget, which uses the openTSNE backend^39^. The option “Preserve global structure” was enabled, which replaces the single-perplexity parameter with a multiscale neighbourhood scheme, allowing the embedding to capture both local similarities and global relationships between spectra. In their t-SNE analysis, Makogon et al.^1^ instead used scikit-learn’s fixed-perplexity implementation (perplexity = 30) without the multiscale option. The multiscale scheme makes t-SNE more comparable to UMAP, which Makogon et al.^1^ found to yield the most compact, well-separated clusters on synthetic EIS spectra.

For each of the four normalization methods and three embedding methods, and for cluster numbers ranging from 2 to 8, linear projections, silhouette plots, hierarchical trees, and clustering metrics were computed, resulting in a total of 84 evaluated cases. The complete set of linear projection maps, silhouette graphs, and dendrograms for all evaluated cases is provided in the *Results/Graphs* folder of the repository (see Data Availability). Here, “linear projection” refers to the two-dimensional plotting space used for visualization, rather than to the mathematical nature of the dimensionality-reduction method, which may be linear (PCA) or nonlinear (t-SNE).

The first criterion to select the optimum clustering configuration was clustering quality, assessed through internal metrics including the silhouette score, Davies–Bouldin index, and Calinski–Harabasz index. These indices jointly reflect the compactness and separability of clusters. To identify the best-performing pipeline across multiple metrics, each clustering configuration was ranked per metric, and the average rank was computed using a Borda^40^ aggregation approach. The minimum inter-centroid distance was calculated but excluded from this ranking, as it systematically decreases with an increasing number of clusters and therefore does not directly reflect clustering quality.

According to the resulting Borda ranking, per-block normalization followed by PCA + t-SNE with six clusters achieved the best average rank, confirming it as the overall best-performing pipeline (Table 1). This configuration exhibited a silhouette score of 0.6235, a Davies–Bouldin index of 0.4673, and a Calinski–Harabasz index of 133.5, reflecting both intra-cluster compactness and inter-cluster distinction. The per-column autoscaled PCA + t-SNE with eight clusters, ranked second, reached the highest Calinski–Harabasz index (158.1) and a similar Davies–Bouldin index (0.472), suggesting stronger overall separation but at the cost of greater fragmentation. The complete list of metric values and corresponding Borda rankings for all evaluated pipelines is provided in the file pipeline_borda_ranking.csv, located in the *Results/Cluster Metrics* folder of the repository (see Data Availability).

Although the dataset originally contained eight mechanistic subgroups, the ranking favored merging two subgroup pairs into six clusters. As shown later by bootstrapping, the six-cluster configuration provided a more robust and mechanistically consistent representation, offering the best balance between numerical clustering quality and physical interpretability.

**Table 1:** Cluster separation metrics across embeddings for the six-cluster solution.

Metric	PCA	PCA + t-SNE	t-SNE	Full spectra
Silhouette score	0.507	0.653	0.625	0.421
Davies–Bouldin index	0.630	0.424	0.464	0.991
Calinski–Harabasz index	62.193	234.39	180.445	25.658
Minimum inter-centroid dist.	0.364	2.242	2.403	6.995

Silhouette score (higher is better), Davies–Bouldin index (lower is better), Calinski–Harabasz index (higher is better), and minimum inter-cluster centroid distance are reported for per-block min–max normalized spectra with dimensionality reduction by PCA, PCA + t-SNE, and t-SNE, as well as for the full spectra without dimensionality reduction.

**Fig. 3:**
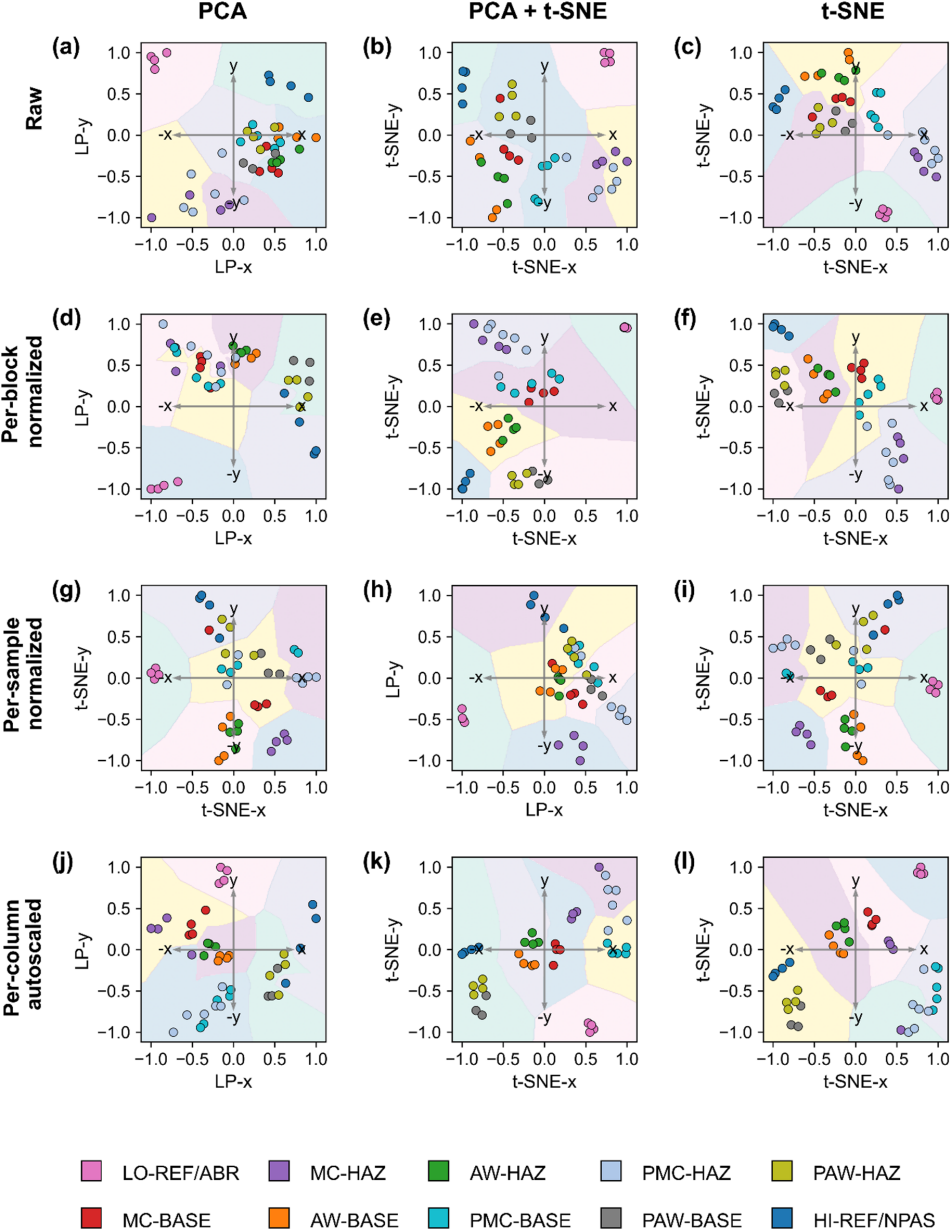
Unsupervised clustering of welded stainless steel impedance spectra in reduced feature spaces for the six-cluster solution. Per-block min-max normalized spectra were projected either linearly by PCA alone (PC1–PC4 arranged in a circular layout) or nonlinearly by t-SNE alone (x and y denote embedding coordinates), or by a PCA + t-SNE pipeline. In the PCA-only projection, the positive y-axis corresponds to PC1, the positive x-axis to PC2, the negative x-axis to PC3, and the negative y-axis to PC4. Panels show: a, raw spectra; b, per-block min–max normalized spectra; c, per-sample min–max normalized spectra; and d, per-column autoscaled spectra. Background regions are obtained by a 1-nearest-neighbor decision map based on the clustered training data (k = 6), revealing distinct groupings. Coloured points represent mechanistic subgroups. Among the tested normalization methods, per-block min–max normalization produced the most compact and well-separated clusters, although with merging of two subgroup pairs. In the PCA + t-SNE pipeline, ABR and NPAS reference subgroups appear at opposite extremes of the projection space.

**Fig. 4:**
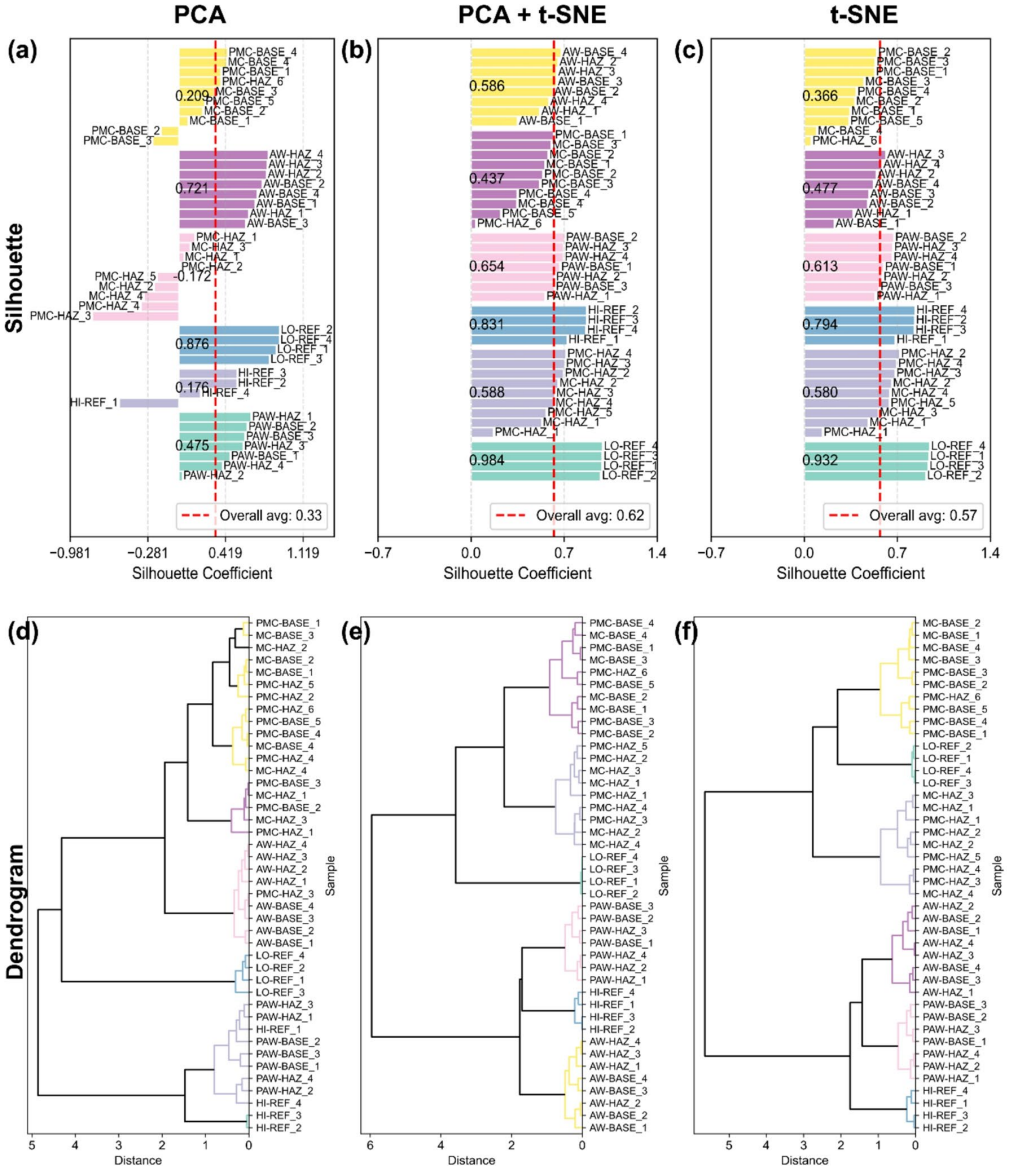
Silhouette plots and hierarchical clustering dendrograms for the six-cluster solution under different dimension-reduction approaches. a, silhouette plots for per-block min–max normalized features. b, hierarchical clustering dendrograms (Euclidean distance, Ward’s linkage) based on the same features. Both analyses were performed for three embeddings: PCA, PCA + t-SNE, and t-SNE.

A comparison of normalization and dimensionality reduction approaches (Table 1; Figs. 3 and 4) shows that both steps significantly affect the resulting clustering structure. Among the tested normalization methods, per-block normalization provided the most consistent clustering behavior, while per-sample and per-column approaches resulted in either reduced contrast between groups or increased within-group variability. Regarding dimensionality reduction, PCA alone preserved global trends but did not always achieve sufficient cluster separation, whereas t-SNE improved local separation but occasionally distorted the overall relationships. The combined PCA + t-SNE approach offered the most balanced outcome, yielding compact and well-separated clusters without excessive fragmentation. When compared with clustering performed directly on the full spectra (Table 1), the use of reduced embeddings led to clearer group boundaries and higher internal cluster metrics, confirming that dimensionality reduction contributes to a more stable and interpretable clustering structure.

### Cluster stability analysis

To assess the stability of the six-cluster structure observed in Fig. 3e (merged subgroups), we performed 100 bootstrap resampling runs and quantified assignment consistency using ARI, NMI, and purity. Internal consistency was evaluated by comparing bootstrap outcomes with the full-sample clustering (Table 2), and external consistency by comparison with the mechanistic subgroups (Table 3). The bootstrap analysis was performed using the *Bootstrap_PCA_t_SNE_workflow.ows* (Code folder) and the merged dataset *input_data_merged.csv* (Input Data folder). Results from 101 runs, including the initial non-bootstrap baseline, are provided in *Results/Bootstrap/cluster_assignments_log.csv*, with summary statistics in *Results/Bootstrap/bootstrap_summary.csv* (see Data Availability).

In the bootstrap procedure, only non-reference subgroups were resampled, and results were averaged with and without the ABR/NPAS references to assess their influence. Stratified and non-stratified resampling produced comparable sample count ranges across configurations (min/median/max numbers of non-reference samples per run: for the 6-cluster pipeline, stratified 17 / 22 / 34 and non-stratified 18 / 22 / 34; for the 8-cluster pipeline, stratified 19 / 23 / 34 and non-stratified 16 / 22 / 34).

Bootstrap resampling confirmed the superior stability of the six-cluster configuration (Per-block + PCA + t-SNE) relative to the eight-cluster alternative (Per-column + PCA + t-SNE). Under stratified sampling, the six-cluster model achieved near-perfect reproducibility, with median ARI = 0.903, NMI = 0.948, purity = 0.968, and Self-ARI = 1.000. Non-stratified resampling slightly reduced these values (median ARI = 0.894, NMI = 0.945, purity = 0.964, Self-ARI = 0.957) but preserved the same cluster topology.

**Table 2:** Internal stability through bootstrap resampling.

Metric	Stratified (median [IQR])	Stratified (mean ± SD)	Stratified (min–max)	Non-stratified (median [IQR])	Non-stratified (mean ± SD)	Non-stratified (min–max)
Per-block + PCA + t-SNE + 6_clusters						
ARI	0.903 [0.893–1.000]	0.915 ± 0.075	0.622–1.000	0.894 [0.718–0.918]	0.833 ± 0.133	0.444–1.000
NMI	0.948 [0.946–1.000]	0.958 ± 0.035	0.828–1.000	0.945 [0.863–0.952]	0.921 ± 0.060	0.710–1.000
Purity	0.968 [0.967–1.000]	0.967 ± 0.041	0.767–1.000	0.964 [0.857–0.969]	0.923 ± 0.069	0.714–1.000
SELF ARI	1.000 [1.000–1.000]	0.972 ± 0.074	0.622–1.000	0.957 [0.781–1.000]	0.885 ± 0.129	0.518–1.000
Per-column + PCA + t-SNE + 8_clusters						
ARI	0.894 [0.718–0.918]	0.833 ± 0.133	0.444–1.000	0.637 [0.595–0.693]	0.642 ± 0.083	0.384–0.854
NMI	0.945 [0.863–0.952]	0.921 ± 0.060	0.710–1.000	0.875 [0.860–0.903]	0.877 ± 0.036	0.765–0.955
Purity	0.964 [0.857–0.969]	0.923 ± 0.069	0.714–1.000	0.769 [0.739–0.800];	0.769 ± 0.050	0.630–0.889
SELF ARI	0.919 [0.869–1.000]	0.922 ± 0.082	0.627–1.000	0.841 [0.780–0.912]	0.836 ± 0.105	0.573–1.000

Distributions of clustering stability metrics (ARI, NMI, purity, and self-ARI) obtained from 100 bootstrap resamplings, where only non-reference subgroups were resampled while clustering was performed on the full dataset. Each bootstrap result was compared with the full, non-bootstrapped clustering (internal stability). Results are presented separately for stratified and non-stratified resampling to assess the influence of sampling strategy on clustering stability.

**Table 3:** External stability through bootstrap resampling without reference samples.

Metric	Stratified (median [IQR])	Stratified (mean ± SD)	Stratified (min–max)	Non-stratified (median [IQR])	Non-stratified (mean ± SD)	Non-stratified (min–max)
Per-block + PCA + t-SNE + 6 clusters (no reference)						
ARI	0.872 [0.860–1.000]	0.899 ± 0.080	0.604–1.000	0.861 [0.763–0.899]	0.840 ± 0.123	0.359–1.000
NMI	0.911 [0.905–1.000]	0.934 ± 0.050	0.807–1.000	0.909 [0.857–0.931]	0.901 ± 0.072	0.606–1.000
Purity	0.958 [0.955–1.000]	0.968 ± 0.030	0.810–1.000	0.957 [0.952–1.000]	0.960 ± 0.040	0.750–1.000
SELF ARI	1.000 [1.000–1.000]	0.976 ± 0.066	0.604–1.000	0.957 [0.781–1.000]	0.885 ± 0.129	0.518–1.000
Per-column + PCA + t-SNE + 8 clusters (no reference)						
ARI	0.567 [0.525–0.630]	0.577 ± 0.088	0.375–0.794	0.545 [0.473–0.631]	0.553 ± 0.101	0.338–0.817
NMI	0.822 [0.809–0.862]	0.832 ± 0.046	0.721–0.931	0.815 [0.774–0.857]	0.816 ± 0.047	0.717–0.934
Purity	0.717 [0.682–0.760]	0.717 ± 0.058	0.571–0.850	0.738 [0.696–0.783]	0.744 ± 0.062	0.609–0.909
SELF ARI	0.911 [0.861–1.000]	0.915 ± 0.094	0.606–1.000	0.846 [0.782–0.911]	0.841 ± 0.109	0.506–1.000

Distributions of clustering stability metrics (ARI, NMI, purity, and self-ARI) obtained from 100 bootstrap resamplings, where only non-reference subgroups were resampled while clustering was performed on the full dataset. For both external and internal stability evaluation, the reference groups (ABR and NPAS) were excluded from the calculation of ARI, NMI, and purity. Each bootstrap result was compared with the mechanistic subgroups (external stability). Results are presented separately for stratified and non-stratified resampling to assess the influence of sampling strategy on clustering stability.

When reference subgroups (ABR, NPAS) were excluded, stability metrics remained equally high, confirming that the clustering structure was not dependent on these boundary states. In contrast, the eight-cluster pipeline exhibited lower reproducibility, with median ARI ≈ 0.64 and purity ≈ 0.77 (all samples), and further decline to 0.57 and 0.72 when excluding references. Despite maintaining moderate NMI (≈ 0.87) and strong Self-ARI (≈ 0.92), the eight-cluster solution showed larger run-to-run dispersion, indicating sensitivity to sampling. These results confirm that the six-cluster configuration offers both higher internal consistency and stronger mechanistic alignment, whereas the eight-cluster model introduces unnecessary fragmentation without improving interpretability.

### Ranking relative passivity via k-level clustering

**Fig. 5:**
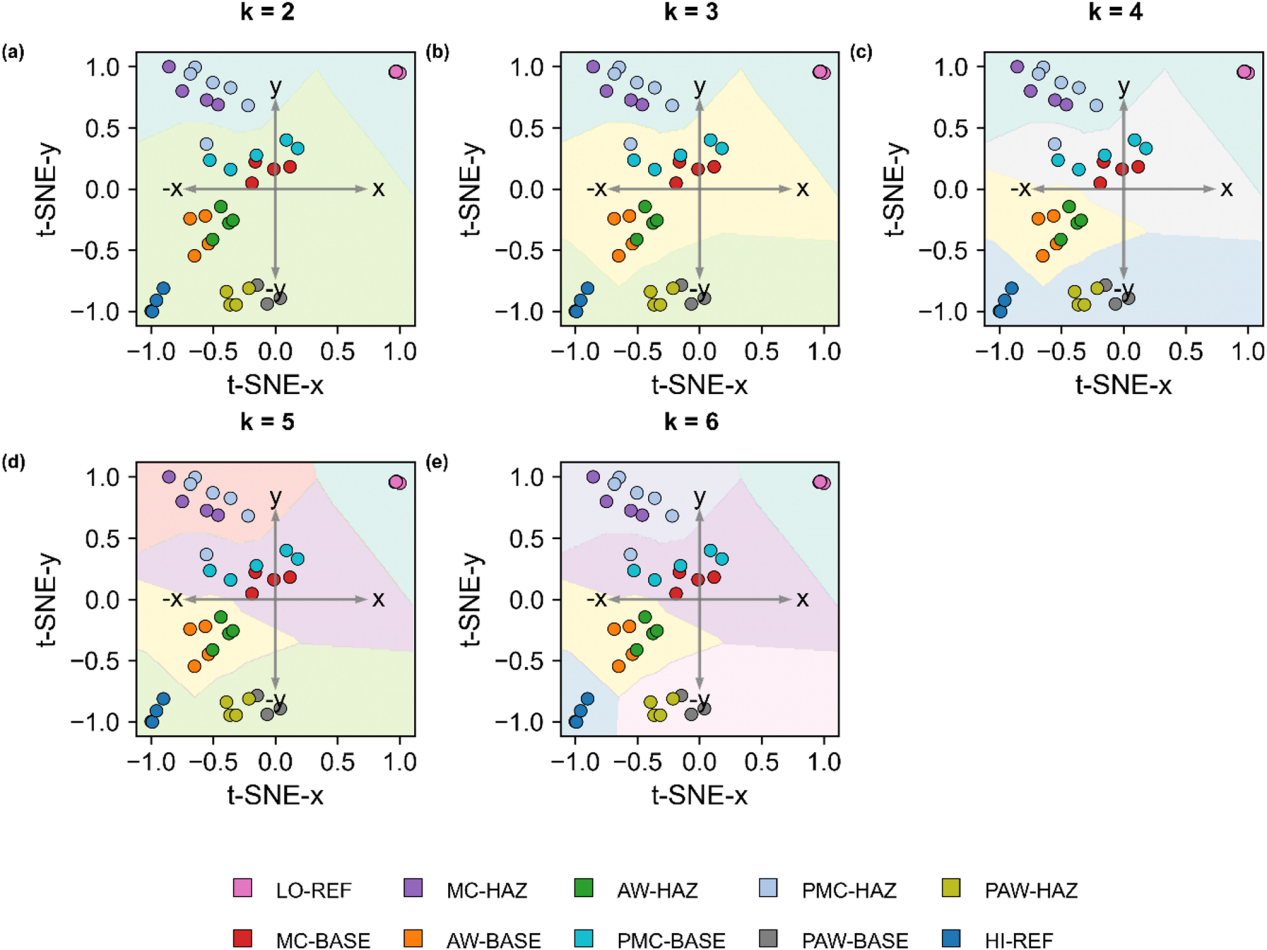
Clustering solutions (k = 2–6) and corresponding passivity scores of 316 L stainless steel. (a) k = 2, (b) k = 3, (c) k = 4, (d) k = 5, (e) k = 6, of the per-lock normalized spectra, projected into the PCA + t-SNE reduced feature space. Coloured points represent mechanistic subgroups, and shaded regions indicate cluster assignment by 1-nearest-neighbor decision mapping. Increasing k progressively refines the passivity distribution, with the six-cluster solution (e) providing the most consistent separation aligned with mechanistic expectations. Although eight mechanistic subgroups were initially defined, the robust six-cluster solution required the merging of two subgroup pairs, which is mechanistically justified.

Figure 5 shows PCA + t-SNE embeddings with cluster numbers ranging from k = 2 to 6. At k = 2 (a), the spectra separate into two broad groups anchored by the ABR (LO-REF) and NPAS (HI-REF) states, defining the passivity extremes. Increasing k progressively refines the partition: at k = 3 (b) NPAS becomes isolated, and at k = 4 (c) intermediate mechanistic subgroups begin to split. Finally, at k = 5 (d) ABR becomes isolated and at k = 6 (e) NPAS separates from the remaining subgroups, so that the six-cluster solution provides the most consistent separation, with ABR and NPAS at opposing corners of the projection space, reflecting their roles as boundary cases and intermediate subgroups arranged in a graded sequence. This progression demonstrates that the structure remains stable across different k values, while the six-cluster partition best captures mechanistically meaningful differences.

From the progressive partitions in Fig. 5, the relative passivity of the samples can be deduced. At *k = 2* (a), the first split isolates MC-HAZ and PMC-HAZ together with ABR (LO-REF), indicating that the heat-affected zones of mechanically cleaned samples are the least passive and not markedly improved by passivation. At *k = 3* (b), NPAS detaches as the top-passivity cluster, while PAW-BASE and PAW-HAZ remain merged, underscoring the beneficial influence of passivation on as-welded samples and their comparatively higher passivity relative to other clusters. At *k = 4* (c), intermediate separation appears, with AW-BASE grouping with AW-HAZ and MC-BASE with PMC-BASE. At *k = 5* (d), MC-HAZ and PMC-HAZ separate from ABR, and at *k = 6* (e), PAW-BASE and PAW-HAZ separate from NPAS. Collectively, these steps place mechanically cleaned HAZ at the low-passivity end, mechanically cleaned BASE and as-welded subsets at intermediate levels, and passivated as-welded samples approaching the nitric-acid-passivated reference.

Figure 5 also reveals distinct effects of surface preparation and post-treatments on the relative resistance of the base metal (BASE) and the heat-affected zone (HAZ). Mechanical cleaning accentuates the spectral contrast between BASE and HAZ; within each region, passivation does not create a separate group (MC-BASE merges with PMC-BASE, and MC-HAZ merges with PMC-HAZ; Fig. 5a–e). This unsupervised grouping indicates that the BASE–HAZ difference outweighs the passivation effect, consistent with abrasion exposing underlying microstructural heterogeneities. The subgroups exhibit only very subtle spectral differences, and separability may be further limited by surface heterogeneity or, as in this case, by differences in prior surface history, rather than by the clustering method itself. These observations agree with reports that weld surface quality strongly influences corrosion resistance^28,41^.

In contrast, as-welded samples are covered by a thermally grown oxide scale (brownish film). This scale masks local heterogeneities, grouping BASE and HAZ together (AW-BASE merges with AW-HAZ, and PAW-BASE merges with PAW-HAZ in Figs. 5a–e) and separating non-passivated from passivated states, with more uniform and higher apparent resistance than mechanically cleaned surfaces. The oxide acts as a stabilizing barrier in the test electrolyte^38^, although such weld oxides can be detrimental under corrosive conditions, serving as initiation sites for localized attack.

## Discussion

In the present study, we introduce a systematic benchmarking workflow for systematic examination of normalization and dimensionality-reduction choices and demonstrate that the pipeline design decisively shapes unsupervised outcomes, including cluster number, structure, and stability. To ensure an unbiased selection of the optimal pipeline, all evaluated combinations of normalization, dimensionality reduction, and cluster number were ranked using a Borda aggregation of internal clustering metrics (silhouette, Davies–Bouldin, and Calinski–Harabasz indices). Using stainless steel as a demonstrator, this multi-criteria ranking identified per-block normalization combined with PCA + t-SNE and six clusters as the overall best-performing configuration, achieving the best balance between compactness, separation, and mechanistic interpretability.

While per-sample normalization and per-column autoscaling are customary in chemometrics workflows and are often used as defaults in EIS + ML studies^2,5,11^, per-block normalization has been discussed mainly for multi-block data^44,45^ and, to our knowledge, has not been systematically applied to impedance spectra. Notably, per-block normalization is also not available as a default in widely used machine-learning workflows^37,46–48^, which emphasize per-sample or per-feature scaling, as a result, most EIS + ML studies to date have relied on these conventional options.

Our benchmarking workflow therefore provides an objective route to integrate quantitative ranking with mechanistic validation. By leveraging unsupervised clustering metrics, the workflow (i) identifies candidate normalization approaches, (ii) evaluates suitable cluster numbers, (iii) compares alternative dimensionality-reduction pipelines, and (iv) recovers a cluster structure consistent with mechanistic expectations for the present system. Using mechanistically anchored linear projections, we further ranked relative passivity across treatments via k-level clustering. The procedure may be transferable to other material systems, provided that normalization strategies, embedding parameters, and reference states are re-evaluated and adjusted for each specific case. Finally, bootstrap resampling confirmed high clustering stability despite the modest sample size, with consistent results obtained both with and without the inclusion of reference subgroups.

The unsupervised workflow presented here is not intended to replace equivalent-circuit fitting or conductivity/permittivity extraction, which provide mechanistic parameters explaining why impedance changes. Instead, the method offers a complementary, model-free classification layer operating directly on raw spectra. While internal clustering metrics quantify numerical separability, physical interpretation in this study relies on mechanistic consistency, i.e., whether clusters correspond to known material states such as BASE vs. HAZ or passivated vs. non-passivated surfaces. The emerging groups reflect differences in spectral shape across frequencies rather than simple magnitude shifts, indicating that the workflow captures electrochemically relevant structure that would not be resolved by single-point metrics such as log|Z| at 0.1 Hz. Once the pipeline is defined for a given dataset, new spectra acquired under comparable experimental conditions can be projected into the same reduced feature space and assigned to the closest cluster automatically, without requiring expert interpretation or equivalent-circuit fitting. This pattern-recognition capability is especially valuable for automated or non-expert applications, where reliable state assignment may be more important than full mechanistic modelling.

## Methods

Machine-learning and clustering workflows were implemented in Orange (version 3.32.24), an open-source visual data mining platform^37^. Built-in widgets were combined with custom Python code through Python Script widgets to extend functionality. All workflow files (.ows), documentation, and instructions for reproduction are provided in the data repository (see Data Availability). The full Orange source code is openly accessible at https://github.com/biolab/orange3.

## Data Availability

All datasets, Orange workflows, supplementary figures and statistical results from this study are provided in a structured OSF repository: 10.17605/OSF.IO/XRAV8.

## References

[1] Makogon, A., Kanoufi, F. & Shkirskiy, V. Is unsupervised dimensionality reduction sufficient to Decode the complexities of electrochemical impedance spectra? *ChemElectroChem* **11**, e202300738 (2024).

[2] Albelda Aparisi, P., Fortes Sánchez, E., Rodrigo, C, Masot Peris, L. . & Laguarda-Miró, N. A rapid electrochemical impedance spectroscopy and sensor-based method for monitoring freeze-damage in tangerines. *IEEE Sens. J.* **21**, 12009–12018 (2021).

[3] Ochandio Fernández, A., Olguín Pinatti, C. A., Peris, M. & Laguarda-Miró, N. Freeze-damage detection in lemons using electrochemical impedance spectroscopy. *Sensors* **19**, 4051 (2019). 10.3390/s19184051 PMC6767336 31546932

[4] Roy, D. & Adhikary, A. Detection of ripening stage of Banganapalle mango using KNN method on PCA-reduced EIS data. In *Proc. IEEE Int. Instrum. Meas. Technol. Conf. (I2MTC)* 1–5 (2023).

[5] Conesa, C., Ibáñez Civera, J., Seguí, L., Fito, P. & Laguarda-Miró, N. An electrochemical impedance spectroscopy system for monitoring pineapple waste saccharification. *Sensors* **16**, 188 (2016). 10.3390/s16020188 PMC4801565 26861317

[6] Minetto, T. A. et al. Identifying adulteration of Raw bovine milk with Urea through electrochemical impedance spectroscopy coupled with chemometric techniques. *Food Chem.* **385**, 132678 (2022). 35290953 10.1016/j.foodchem.2022.132678

[7] de Magalhães, J. B. et al. Evaluating adulteration of commercial extra Virgin Olive oil with Canola and sunflower oils through electrochemical impedance spectroscopy. *Food Bioprocess. Technol.* **17**, 2805–2817 (2024).

[8] Zhu, Z., Geng, Y., Wang, Y. & Monitoring single *S. cerevisiae* cells with multifrequency electrical impedance spectroscopy in an electrode-integrated microfluidic device. *Methods Mol. Biol.* **2189**, 105–118 (2021). 10.1007/978-1-0716-0822-7_9 33180297

[9] Yavarinasab, A. et al. A selective polypyrrole-based sub-ppm impedimetric sensor for the detection of dissolved hydrogen sulfide and ammonia in a mixture. *J Hazard. Mater.* **416**, 125892 (2021). 34492830 10.1016/j.jhazmat.2021.125892

[10] Kumar, R. et al. A simple electronic tongue. *Sens. Actuators B Chem.* **171–172**, 1046–1053 (2012).

[11] Miszczyk, A. & Darowicki, K. Multispectral impedance quality testing of coil-coating system using principal component analysis. *Prog Org. Coat.* **69**, 330–334 (2010).

[12] Zia, A. I. et al. Post annealing performance evaluation of printable interdigital capacitive sensors by principal component analysis. *IEEE Sens. J.* **15**, 3110–3118 (2015).

[13] Raj, G. C. A. et al. Polymer-based virtual sensor array leveraging fringing field capacitance for VOC detection. *Proc. IEEE Sens.* **2023**, 1–4 (2023).

[14] Park, S. & Kim, K. H. Principal component analysis implementation for signal processing of electrochemical impedance spectroscopy in the detection of fake fingerprints. In *Proc. Int. Conf. Artif. Intell. Inf. Commun. (ICAIIC)* 411–415 (2021).

[15] Su, Z. P. et al. Modeling and health feature extraction method for lithium-ion batteries state of health Estimation by distribution of relaxation times. *J. Energy Storage Part. A*. **90**, 111770 (2024).

[16] Penjuru, N. M. H. et al. Machine learning aided predictions for capacity fade of Li-ion batteries. *J. Electrochem. Soc.* **169**, 050535 (2022).

[17] Chang, C. et al. Lithium-ion battery state of health estimation based on electrochemical impedance spectroscopy and cuckoo search algorithm optimized Elman neural network. *J. Electrochem. Energy Convers. Storage*. **19**, 030912 (2022).

[18] Chen, J. et al. Time-lapse electrochemical impedance detection of bacteria proliferation for accurate antibiotic evaluation. *IEEE Sens. J.* **22**, 5504–5513 (2022).

[19] Obregon, J. et al. Convolutional autoencoder-based SOH Estimation of lithium-ion batteries using electrochemical impedance spectroscopy. *J. Energy Storage*. **60**, 106680 (2023).

[20] Zhang, L. et al. Estimation of lithium-ion battery health state using enhanced variational autoencoder and bidirectional gated recurrent unit based on electrochemical impedance spectroscopy. *J. Power Sources*. **655**, 237957 (2025).

[21] Yuan, J. et al. Unsupervised feature extraction for lithium-ion battery electrochemical impedance spectroscopy and capacity Estimation using deep learning method. *Electrochim. Acta*. **498**, 144694 (2024).

[22] Meng, J. et al. A domain-adversarial neural network for transferable lithium-ion battery state-of-health Estimation. *IEEE Trans. Transp. Electrific*. **11**, 3 (2025).

[23] Lou, C. et al. Extract features from lithium-ion battery electrochemical impedance spectra and estimate state of health based on improved convolutional autoencoder–temporal convolutional network. *Ionics* **31**, 4261–4279 (2025).

[24] Doonyapisut, D. et al. Deep generative learning for exploration in large electrochemical impedance dataset. *Eng. Appl. Artif. Intell.* **126** (Part C), 107027 (2023).

[25] Soares, C. et al. Electrochemical impedance spectroscopy characterization of beverages. *Food Chem.* **302**, 125345 (2020). 31445377 10.1016/j.foodchem.2019.125345

[26] Sun, J. et al. Inquiry into the appropriate data preprocessing of electrochemical impedance spectroscopy for machine learning. *J. Phys. Chem. C*. **129**, 1044–1051 (2025).

[27] Coelho, L. B. et al. Identifying stable pitting pathways in 316 L stainless steel via fractal-inspired PCA-based clustering. *Npj Mater. Degrad.* **9**, 42 (2025).

[28] Kurtz, M. A. et al. Oxide degradation precedes additively manufactured Ti-6Al-4V selective dissolution: an unsupervised machine learning correlation of impedance and dissolution compared to Ti-29Nb-21Zr. *J. Biomed. Mater. Res. A*. **112**, 1250–1264 (2024). 37877770 10.1002/jbm.a.37632

[29] Kurtz, M. A. et al. Deep neural network predicts Ti-6Al-4V dissolution state using near-field impedance spectra. *Adv. Funct. Mater.* **34**, 2308932 (2024).

[30] Kurtz, M. A. et al. Predicting corrosion damage in the human body using artificial intelligence: in vitro progress and future applications. *Orthop. Clin. N Am.* **54**, 169–192 (2023). 10.1016/j.ocl.2022.11.004 36894290

[31] Jolliffe, I. T. *Principal Component Analysis. Springer Series in Statistics* (Springer, 2002).

[32] van der Maaten, L. & Hinton, G. Visualizing data using t-SNE. *J. Mach. Learn. Res.* **9**, 2579–2605 (2008).

[33] Rousseeuw, P. J. Silhouettes: a graphical aid to the interpretation and validation of cluster analysis. *J. Comput. Appl. Math.* **20**, 53–65 (1987).

[34] Davies, D. L. & Bouldin, D. W. A cluster separation measure. *IEEE Trans. Pattern Anal. Mach. Intell.* **PAMI-1**, 224–227 (1979). 21868852

[35] Caliński, T. & Harabasz, J. A dendrite method for cluster analysis. *Commun. Stat.* **3**, 1–27 (1974).

[36] Vinh, N. X., Epps, J. & Bailey, J. Information theoretic measures for clusterings comparison: variants, properties, normalization and correction for chance. *J. Mach. Learn. Res.* **11**, 2837–2854 (2010).

[37] Demsar, J. et al. Orange: data mining toolbox in python. *J. Mach. Learn. Res.* **14**, 2349–2353 (2013).

[38] Bera, I., Bašurić, I., Šoić, I. & Martinez, S. Investigating the impact of post-weld cleaning and passivation on the performance of austenitic stainless steel using an EIS paste-electrolyte cell. *J. Solid State Electrochem.* **28**, 2827–2836 (2024).

[39] Poličar, P. G., Stražar, M. & Zupan, B. openTSNE: A modular python library for t-SNE dimensionality reduction and embedding. *J. Stat. Softw.* **109**, 1–30 (2024).

[40] Barak, S., Abessi, M. & Modarres, M. Evaluation and selection of clustering methods using a hybrid group MCDM. *Expert Syst. Appl.* **138**, 112817 (2019).

[41] Łastowska, O., Jurczak, W. & Starosta, R. Influence of surface quality on corrosion resistance of stainless steel and aluminum alloy butt welds after innovative finishing. *Sci. Rep.* **15**, 27576 (2025). 40730681 10.1038/s41598-025-13473-7 PMC12307888

[42] Cao, L. et al. Effect of surface roughness on corrosion behaviour of stainless steel in supercritical CO₂. *J. Mater. Res. Technol.* **36**, 4946–4954 (2025).

[43] Brajković, T., Juraga, I. & Šimunović, V. Influence of surface treatment on corrosion resistance of Cr-Ni steel. *Eng. Rev.* **33**, 129–134 (2013).

[44] Westerhuis, J. A., Kourti, T. & MacGregor, J. F. Analysis of multiblock and hierarchical PCA and PLS models. *J. Chemometrics*. **12**, 301–321 (1998).

[45] Smilde, A. K., Bro, R. & Geladi, P. *Multi-way Analysis: Applications in the Chemical Sciences* (Wiley, 2004).

[46] Pedregosa et al. Scikit-learn: machine learning in python. *J. Mach. Learn. Res.* **12**, 2825–2830 (2011).

[47] Abadi et al. TensorFlow: a system for large-scale machine learning. In *OSDI’16: 12th USENIX Symposium on Operating Systems Design and Implementation* 265–283 (2016).

[48] Eigenvector Research Inc. *SOLO and PLS_Toolbox Documentation* (Eigenvector Research, 2025).

